# Postnatal Development of the Murine Notochord Remnants Quantified by High-resolution Contrast-enhanced MicroCT

**DOI:** 10.1038/s41598-017-13446-5

**Published:** 2017-10-17

**Authors:** Sameer Bhalla, Kevin H. Lin, Simon Y. Tang

**Affiliations:** 1 Department of Biology, Washington University in St. Louis, Missouri, 63105 USA; 2 Department of Orthopaedic Surgery, Washington University in St. Louis, Missouri, 63110 USA; 3 Department of Biomedical Engineering, Washington University in St. Louis, Missouri, 63105 USA; 4 Department of Materials Science and Mechanical Engineering, Washington University in St. Louis, Missouri, 63105 USA

## Abstract

The notochord gives rise to spinal segments during development, and it becomes embedded within the nucleus pulposus of the intervertebral disc (IVD) during maturation. The disruption of the notochord band has been observed with IVD degeneration. Since the mechanical competence of the IVD relies on its structural constituents, defining the structure of the notochord during aging is critical for investigations relating to IVD function and homeostasis. The assessment and imaging of the notochord has classically relied on histological techniques, which introduces sectioning artifacts during preparation and spatial biases. Magnetic resonance imaging (MRI) does not offer sufficient resolution to discriminate the notochord from the surrounding the nucleus pulposus, especially in murine models. Current X-ray based computed tomography systems provide imaging resolutions down to the single- and sub- micron scales, and when coupled with contrast-enhancing agents, enable the high-resolution three-dimensional imaging of relatively small features. Utilizing phosphomolybdic acid to preferentially bind to collagen cationic domains, we describe the structure of the notochord remnants with aging in the lumbar IVDs of BALB/c mice. These results provide a highly quantitative and sensitive approach to monitoring the IVD during postnatal development.

## Introduction

The notochord is a critical feature for all vertebrate organisms that forms from mesodermic origins during embryogenesis^1,2^, and it provides developmental cues for the patterning and formation of key anatomical structures including the ventral neural tube, the axial skeleton, and the intervertebral disc (IVD)^3–5^. Although remnants of the notochordal tissue become embedded in the nucleus pulposus of the IVD during postnatal maturation, the notochord cells nevertheless maintain a unique phenotype and extracellular matrix^1–5^. The structural progression of the extracellular matrix surrounding notochordal cells during aging remains relatively unknown.

The IVD is a fibrocartilaginous joint that transmits and dampens loads, and provides mobility to the spine. Each IVD lies between two adjacent vertebrae in the vertebral column and consists of the superior and inferior cartilaginous end plates, the outer annulus fibrosus (AF), and the inner nucleus pulposus (NP)^6,7^. In mature organisms, the notochord (NC) has observed to be a nondiscrete structure scattered through the center of the NP, and has implications in the regenerative capacity of the IVD^5,8,9^. Understanding the structure of the notochord is an important step in defining the mechanical and mechanobiologic roles of this tissue in IVD homeostasis and maintenance.

Structural studies of the  IVD typically involve histological techniques or electron microscopy^10,11^. Histological approaches such as whole-mount offer insightful information regarding the spatial postnatal development at the macro-level, and sectioning allow the identification of molecular expression with tissue-specific context^12–14^. Confocal and electron microscopy provide detailed information within a very localized area^11^. Yet these approaches do not allow a full three-dimensional assessment as they require the destructive preparation of the sample. Magnetic resonance imaging (MRI) offers the opportunity to nondestructively image the IVD but with limited resolutions for fine structures such as the notochord^15^.  XRay computed tomography techniques offer very high resolution detection of radio-opaque  features, and there are a number of approaches that can enhance the ability to resolve other features such as phase-contrast, Talbot-Lau interferometry, and contrast enhancement^16–22^. High-resolution contrast-enhanced micro-computed tomography (microCT) approaches that leverage contrast agents have been used to enhance the attenuation of the murine IVD^16,17^.

In this study, Phosphomolybdic Acid (PMA), which targets collagen residues^23,24^, was utilized in combination with 1- and 6- micron spatial resolution microCT to assess the postnatal development of mouse lumbar intervertebral discs up to a year of age. Moreover, we specifically report quantitative three-dimensional metrics: the notochord surface area to volume ratio (S.a/V), which defines the structure of the notochord, and the notochord volume. These data provide important normative data for the three-dimensional notochord structure with aging, and it will be valuable in future studies of intervertebral disc disease and degeneration.

## Results

**Figure 1:**
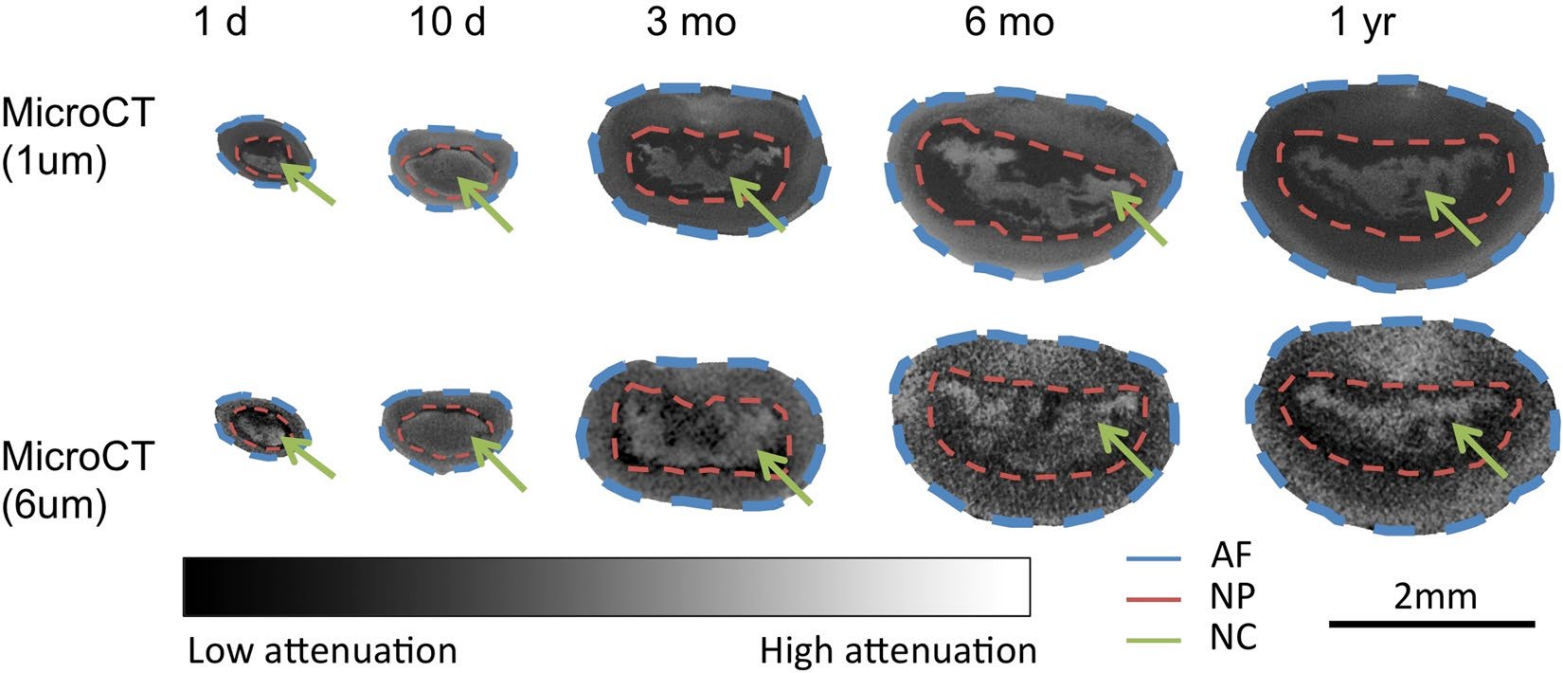
Grayscale images of transverse sections from murine intervertebral discs across different ages. The darker shades reflect lowly attenuating regions and, and the lighter shades reflect highly attenuating regions. The blue dashed line outlines the annulus fibrosus (AF) region, the red dashed line outlines the nucleus pulposus (NP) region, and the green arrows indicate the notochordal (NC) remnants. The notochord structure is highly attenuating from the PMA chelation of the collagen residues. The AF and NC tissues attenuate higher than the NP, allowing for segmentation of three distinct compartments. At early timepoints, the NC is a nondiscrete structure that spans across a large area of the NP. With age, the NC becomes more discrete within the middle of the NP and grows in size.

**Figure 2:**
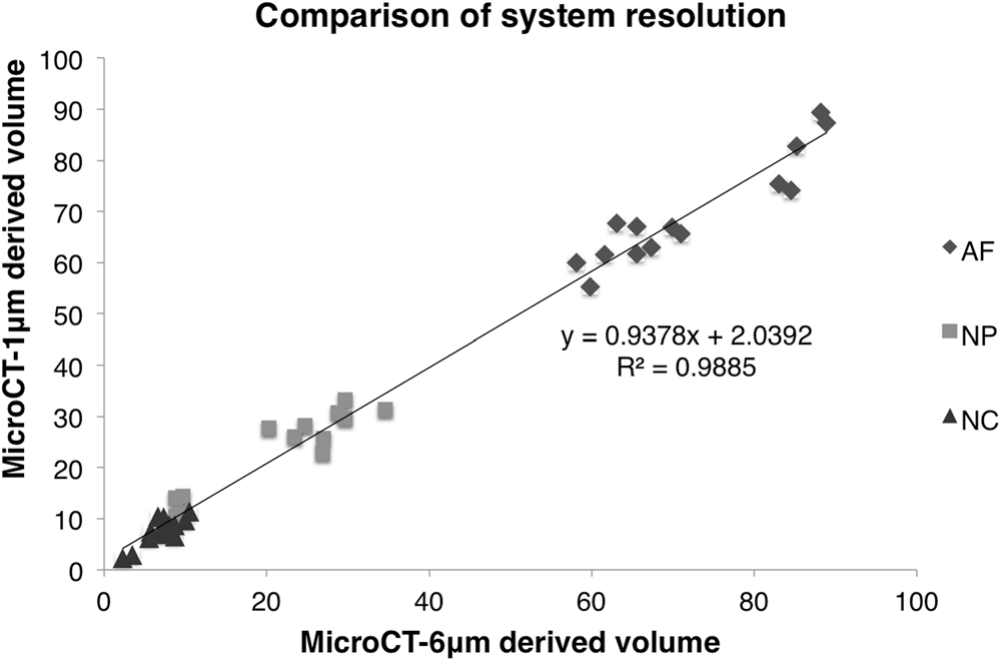
A comparison of the computed AF, NP, and NC volume percentages obtained from the microCT-1 µm and microCT-6 µm systems.

Phosphomolybdic acid-stained functional spinal units (FSUs) revealed increased attenuation of the notochordal tissues across all samples (Fig. 1). The Intraclass correlation coefficient (ICC) confirmed that the contouring and segmentation for the disc was reproducible (Table 1). The microCT-1µm and microCT-6 µm-derived IVD compartment volumes were statistically indistinguishable (paired t-test, p = 0.99) and highly correlated (Pearson’s correlation, p < 0.001, r^2^ = 0.99), suggesting that scanning at 6 µm spatial resolution is a reasonable compromise to measure the notochord volume (Fig. 2; Figure S1). Examination of the correlations in the individual AF, NP, and NC compartments show that although while the 1µm- and 6 µm- microCT data were highly correlated for the AF (Pearson’s correlation, p < 0.001, r^2^ = 0.87) and the NP compartments (Pearson’s correlation, p < 0.01, r^2^ = 0.89), the NC compartment correlation resulted in significant, but less correlated values (Pearson’s correlation, p < 0.05, r^2^ = 0.67), suggesting that there are trade-offs in accuracy in the 6 µm-resolution scans. An examination of the grayscale transverse sections confirms that the images obtained at the 1 µm resolution reveals more detail particularly of the notochord and the collagen-rich extra-cellular matrix (Fig. 1).

**Table 1:** The intraclass correlation (ICC) for each calculated value is above 0.95, confirming that contouring approach of the whole disc, nucleus, and setting a lower-threshold for the notochord tissue is highly reliable.

	ICC	95% confidence interval	
		Lower Bound	Upper Bound
Total Volume	0.969	0.727	0.999
AF Volume	0.955	0.627	0.999
NP Volume	0.978	0.795	0.999
NC Volume	0.985	0.857	0.999
NC Area	0.989	0.888	0.999

The ICC upper and lower bounds suggest a high degree of reproducibility^26^.

**Figure 3:**
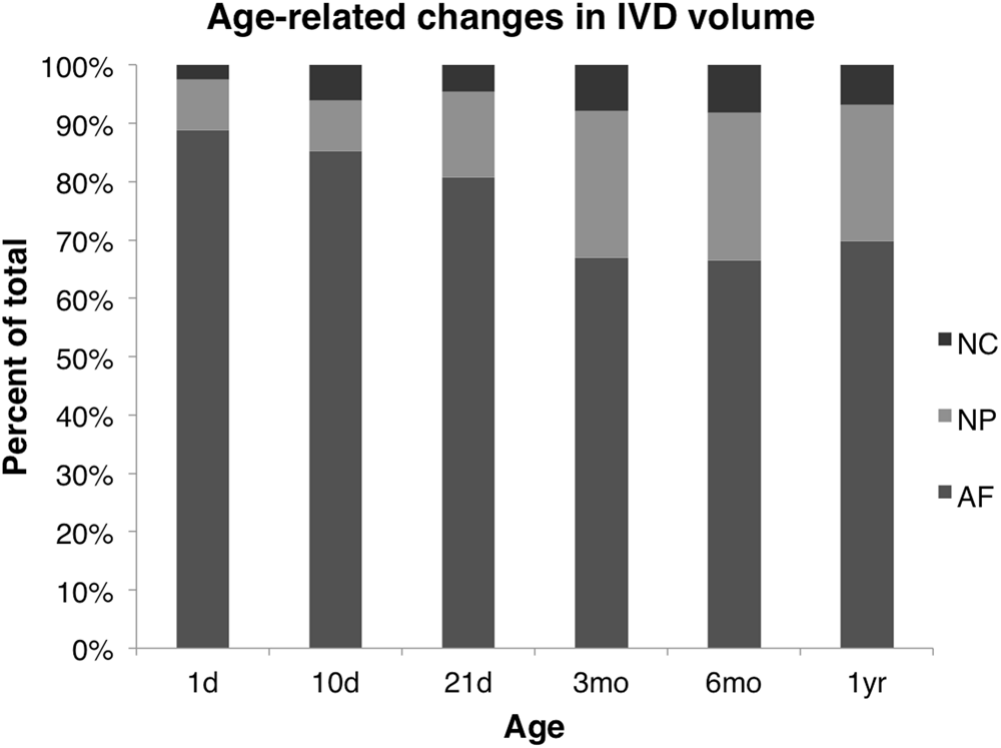
Three compartments of the murine intervertebral disc expressed as a percentage of the total IVD volume, generated using the microCT-6 µm images. Annulus fibrosus (AF), the inner nucleus pulposus (NP), and the notochord (NC) percentages are normalized to the respective discs and averaged within the age group.

**Figure 4:**
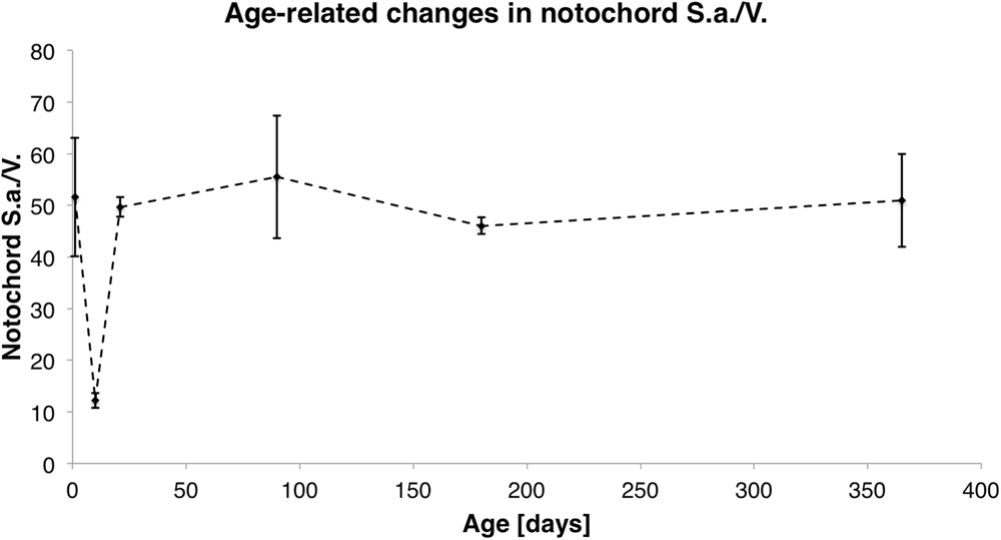
The notochord Surface area/Volume (S.a/V.) ratio provides insight to the change in the general shape of the notochord tissue with age. High S.a/V. indicates a more diffuse structure, while a low S.a/V. suggests that the notochord is more block like.

**Figure 5:**
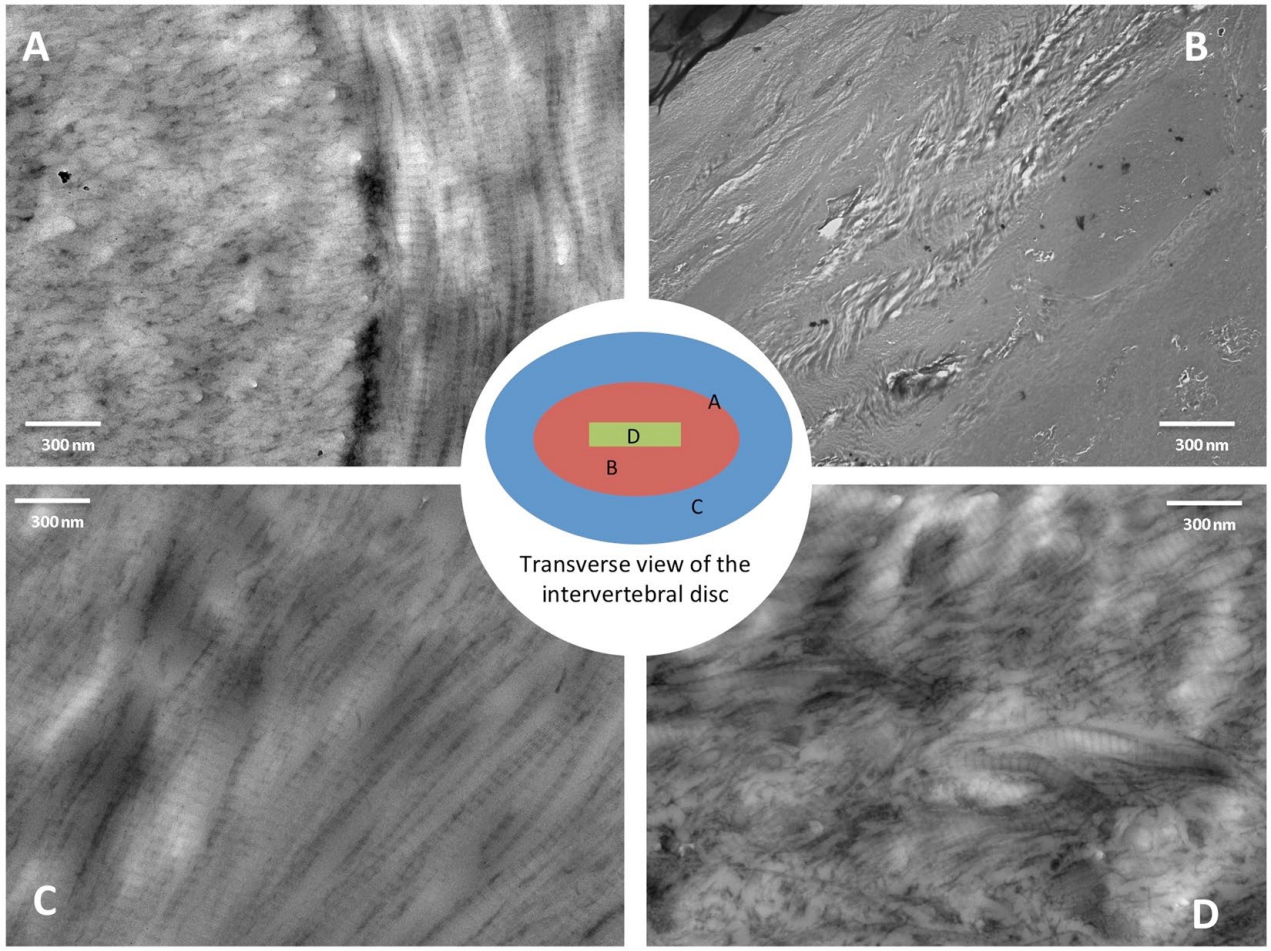
Transmission electron micrographs from the transverse sections of the PMA-stained intervertebral discs. (**A**) The annulus-nucleus interface confirms the abundance of collagen fibers in the annulus fibrosus with a transition in PMA-intensity and nano-scale structure towards the nucleus pulposus. (**B**) Within the nucleus pulposus, there’s a lack of PMA staining that is consistent with very low presence of collagen in this region. (**C**) The annulus fibrosus is collagen rich and can be confirmed by both the PMA-staining and the characteristic banding of collagens. (**D**) The notochordal remnants region appear to contain small amounts of collagen.

**Figure 6:**
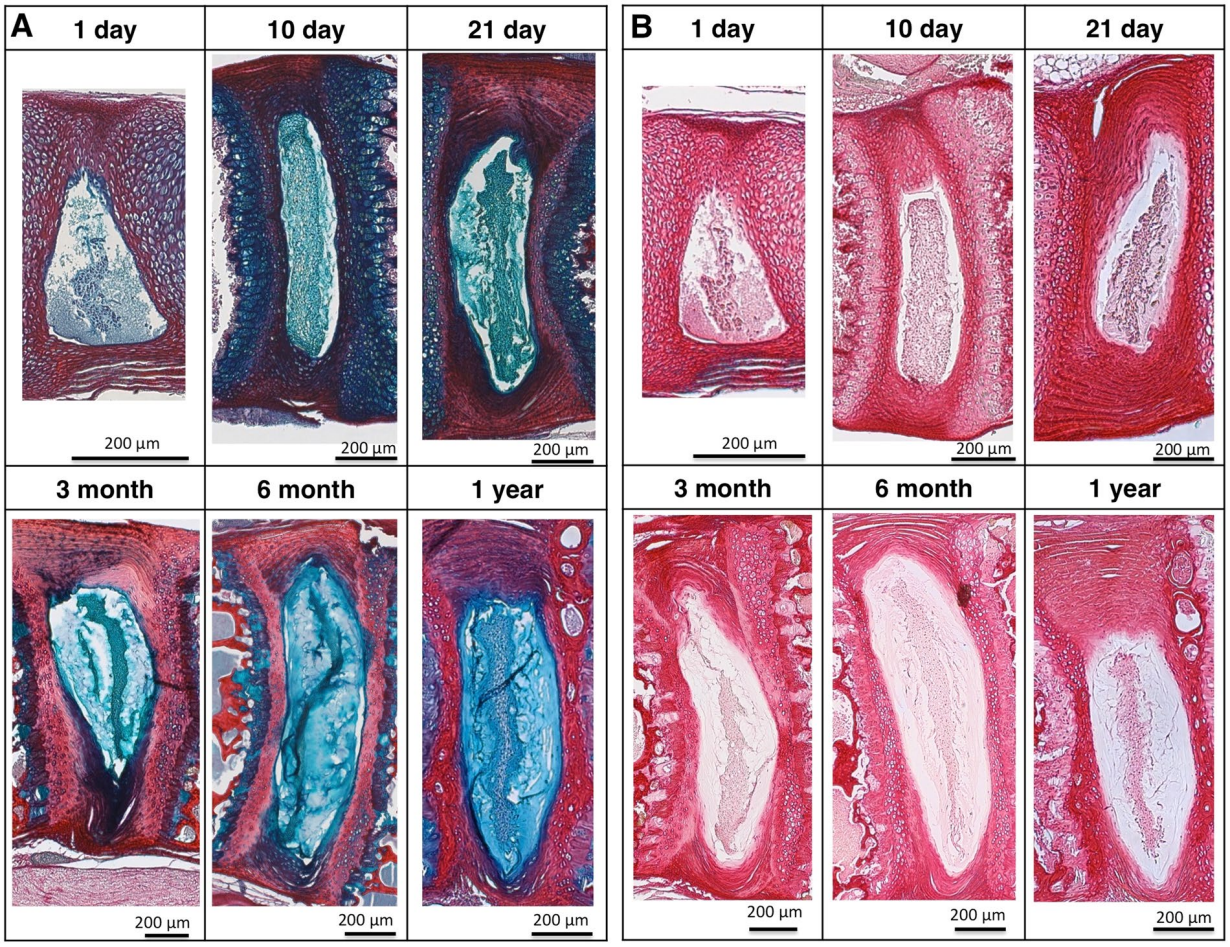
Histological staining of intervertebral discs in the sagittal orientation (**A**) Picrosirius red; and (**B**) Alcian blue and picrosirius red. Picrosirius red stains for collagen and alcian blue stains for glycosaminoglycans.

During aging, the notochord expands in volume but remains a relatively small percentage of the total IVD volume (Fig. 3; ANOVA, p < 0.0001). Additionally, the notochord surface area to volume ratio, which provides a measure for the morphology of the notochord, dramatically changes at different stages of postnatal development (Fig. 4; ANOVA, p < 0.0001). High S.a/V. indicates that there are more surfaces than volume of notochord suggesting a more diffuse structure, while a low S.a/V. suggests that the notochord is more block-like^25^. At the 1-day time point, the S.a./V. ratio is high, confirming the visually apparent nondiscrete shape and diffuse structure. As the notochord matures to the 10-day time point, it becomes a more discrete volume and the significant decrease of notochord S.a./V. indicates that it has condensed in structural morphology despite of the increasing volume (ANOVA Tukey’s LSD, p < 0.05). With aging, the notochord concomitantly increases in volume but maintains its S.a./V. ratio suggesting that the structure enlarges but maintains its morphology. Transmission electron micrographs confirm that PMA-stained collagen structures throughout the notochord and the annulus fibrosus  (Fig. 5). Picrosirius red staining confirms the presence of collagen in the notochord coinciding with the alcian blue-stained glycosaminoglycans in the nucleus pulposus (Fig. 6).

## Discussion

We developed a novel technique to accurately quantify the volume and surface area changes of notochord tissue in the post-natal mouse IVDs. The phosphomolybdic acid contrast-enhanced microCT imaging process is a nondestructive way to image IVDs without introducing spatial biases that are susceptible in histology. By introducing a reliable method to quantify notochord tissue within the IVD, we can begin to formulate a clearer picture of the role notochord tissue and its remnants during postnatal maturation. The histology by picrosirius red confirms the presence of collagen in the notochordal tissue (Fig. 6A), and this persists up to the one-year time point^8,12^. The alcian blue confirms the presence of increasing glycosaminoglycans during the maturation of the nucleus pulpolsus (Fig. 6B). The nondestructive assessment here also allows histological preparation and analyses to be conducted after the microCT imaging. Although histology provides high quality images of the tissue, histological preparations of the IVD are highly susceptible to tearing, contraction, and artifacts, especially within the notochordal remnant space^12,27^. This was  also observed in our own histology images (Fig. 6). In contrast, the microCT images revealed a notochord structure with greater nuance and details than the planar two-dimensional observations. Furthermore, the three-dimensional data allows characterizing quantitative changes of the notochord in aging and disease^5,28^. The loss of the notochord cells and alterations in structure have been implicated in disc degeneration^29^. With low back pain being a prevalent public health problem with high public health costs^30,31^, an improved understanding of the role of the notochord is critical for investigatingdisease mechanisms and developing regenerative strategies for low back pain and IVD degeneration. As mouse models are commonly used for mechanistic investigations for the intervertebral disc, it is prudent to understand the murine notochord in these contexts. The notochord cells and the pericellular matrix may also have a mechanbiologic role in the IVD that allow it to respond to the applied stresses and strains in development, aging, and disease^13,32^. Our study gives a unique opportunity to provide highly accurate three-dimensional data for modeling studies that would elucidate the mechanobiology of the notochord. Notochordal cells express high levels of collagen during expansion of the nucleus pulposus ^8,33,34^, and these cells remain embedded in within the nucleus pulposus while retaining their cell-specific characteristics^12–14,35^. Thus, these cells may continue to express collagen resulting in the accumulation of a collagen-rich band observed here. We and others have observed that this collagen-rich notochordal band becomes disrupted during the degeneration and inflammation of murine intervertebral discs^27,36^.

The approach of contrast-enhanced microCT is versatile and multiple contrast agents can be used to highlight different tissues across different time points^16,17^. Although PMA can only be applied to *ex vivo* tissues, other contrast agents such as Ioversol can be applied to living bioactive systems. For example, Ioversol imaging can be done longitudinally during the course of organ culture and then PMA can be utilized at the terminal time point to provide both longitudinal and cross sectional comparisons of the IVD and the notochord^16,17,27^. To ascertain the tradeoffs in resolution and subsequent time and cost, we utilized two microCT systems at two different resolutions. Our results suggest that assessing the IVD at a 6 µm resolution for animals older than 21-days may be sufficient for resolving the IVD microstructures. The notochord tissue appears as a scattered nondiscrete structure one day after birth, then transitions to a more discrete volume with a condensed structural morphology after 10 days. At early time points, the disc consists mainly of a large AF and a small NP and NC. With age, the NC grows as a function of the NP to take up a larger percentage of the whole disc. The increasing size of the notochord suggests that it may have important mechanical roles as a structure within the IVD. It is worth noting that large variations observed in the 1-day old mice may be due to the imprecise dating of the animals and the rapid developmental changes that occur early in life. For example, a 16-hour old mice and a 32-hour old mice are both classified as 1-day old mice. Furthermore, there appears to be a transition in shape of the notochord between 10- and 21- days, and this warrants further investigation.

Other X-ray based techniques may also reveal novel biological insights. For example, it is possible to detect pathologic states during atherosclerosis with the potential for clinical implementation^20^. In tissues where flow permits differential phases, vessel structures can be resolved^19^. The contrast-enhanced microCT technique here generates three-dimensional images of the IVD including the notochord, eliminates preparation errors and spatial bias, and allows a more accurate examination of the IVD’s mechanobiological properties.

## Methods and Materials

### Animals, tissues preparation, and contrast-agent

We obtained wild-type BALB/c mice at  ages 1-day, 10-day, 21-day, 3-months, 6- months, and 1-year (n = 3 at each age). Gender of the animals was randomized in the sample distribution. Prior to dissection, the mice were euthanized with CO_2_ overdose for 7 minutes at a flow rate of 2.5–3 L/min. Manual decapitation was also performed after CO_2_ overdose in young animals. Mice were stored in −20 °C freezer until ready to dissect. All procedures were done with approval from the Washington University Institutional Animal Care and Use Committee. In addition, all experimental procedures were performed in accordance with relevant guidelines and regulations.

**Figure 7:**
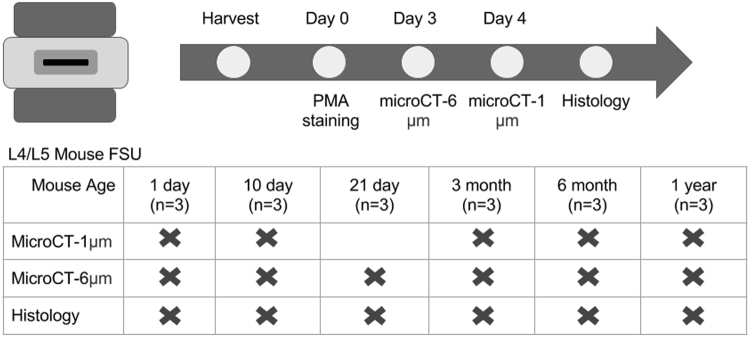
Workflow of the experimental design. A total of 18 L4/L5 FSUs were taken from BALB/c mice. Following imaging in the respective microCT systems , the samples were decalcified and then sectioned for histology.

Using a #11 blade scalpel, a vertical cut was made on the dorsal surface of the mouse carcass from the neck to tail to expose the body cavity. Cuts were made from the L1 vertebrae to the L6 vertebrae on both sides of the vertebral column. Using dissection scissors and forceps, the L1-L6 vertebrae were isolated and removed from the body cavity. Using fine dissection scissors, extra soft tissue surrounding the spine was removed. Next, the spine was dissected into a functional spinal unit (FSU) consisting of a vertebrae-disc-vertebrae structure at the L4/L5 IVD. The FSUs were placed in a 1.5 mL microcentrifuge tube that contained 1 mL of 5% Phosphomolybdic Acid (PMA) (Sigma HT153) solution in DI water. The FSU remained in solution for 72 hours at 20°C. Following the incubation period, the FSU was wrapped in gauze, inserted into a 1.5 mL microcentrifuge tube, and imaged using the microCT systems. A workflow for the samples is shown in Fig. 7.

### Contrast-enhanced Micro-Computed Tomography (CT)

Because high-resolution microCT imaging at 1- micron spatial resolution is computationally and resource intensive, we compared scans from two microCT systems in order to determine whether it is feasible to accurately ascertain the IVD microstructures using a more coarse resolution. Thus, two microCT systems were utilized for this study: The Scanco µCT 40 (Scanco Medical, CH) (microCT-6 µm) system which was operated at a peak of 45 keV, 177 µA, 8 W, 6.0 µm voxel size, and 300 ms integration; and the Zeiss Xradia 520 Versa (Zeiss Group, Germany) (microCT-1µm) which was operated at a peak of 5 keV, 4 W, 7 s exposure, with a 4X objective. Each sample underwent both the microCT-1 µm and microCT-6 µm scans after PMA staining, with the exception of the 21-day samples which were not available for the microCT-1µm analyses. All imaging analyses were conducted independently on both scans from each sample.

### Semi-automated segmentation and volumetric analyses

The microCT scan data were exported as DICOM files to custom MATLAB-based image processing scripts for quantitation of whole disc, NP, and NC volumes. Prior to the analysis, the microCT-6 µm scans were scaled to match the microCT-1µm scans using a polynomial interpolation. Then, a median filter with radius = 5 was applied to all images. For segmentation of the disc, two sets of contours were manually drawn: one contour around the outer edge of the AF and one contour at the AF/NP border. Contours were drawn every 30 slices and then morphed using a linear interpolation into masks representing the whole disc and NP. A third mask of the NC was generated by thresholding the NC tissue due to its high attenuation and irregular shape.

Volumes of the whole disc, NP, and NC are calculated from the total number of voxels enclosed by the respective masks.  AF volume was computed from the difference of the Whold Disc volume and the NP volume.  Volumes of the NP and NC were normalized to a percentage of total disc volume to facilitate comparison between different IVD dimensions across the various timepoints. In addition, the surface area to volume ratio (S.a./V.) for the NC was calculated as a metric of the NC shape. Finally, following each analysis, a 3D image showing all three compartments of the disc is generated (Fig. S1).

### Transmission Electron Microscopy

The FSU segments were dehydrated in a graded ethanol series (50%, 70%, 90%, 100%, 100%) for 10 minutes at each step. Following dehydration, the samples were then embedded into a LR White Hard resin with microwave-assisted infiltration and cured at 60 °C for 24 hours. The tissue was sagittally mounted and bone segments were trimmed away leaving only the disk behind. Once trimmed, 100 nm thick sections were then taken transversely and examined without any counterstaining on a TEM (JEOL JEM-1400 Plus, Tokyo, Japan) at 80 KeV. Several regions from the transverse sections were imaged.

### Histology

The samples were fixed with 10% Neutral Buffered Formalin for 24 hours and decalcified with Immunocal (1414-1, StatLab) solution for 72 hours. Samples were then dehydrated in ethanol and embedded in paraffin. Samples were sliced with a 10 µm thickness in the sagittal plane using a microtome and then applied to glass slides.

Sections were deparaffinized and rehydrated to water. Then, sections were stained in 1% alcian blue solution (1 g Alcian Blue 8GX, (SC-214517A, Santa Cruz Biotech), 3 mL glacial acetic acid (Fisher A35500) and 97 mL DI water) for 30 minutes. The sample was rinsed in tap water for 2 minutes and stained in picrosirius red (0.1 g sirius red F3B (CI 35780, Direct red 80) (Sigma 365548), and/or combined with 100 mL saturated aqueous Picric acid (Sigma P6744) for 2 hours. Following staining, the sections were rinsed with 0.01 N HCl (Fisher SA621) for 2 minutes, dehydrated, cleared, mounted, and coverslipped.

### Statistics

An intraclass correlation (ICC) was conducted to assess the reliability of the contouring and segmentation from three independent measurements. The operator is blinded to the age and utilized scanner. Paired t-tests were conducted to detect the differences between the data from the different scanners. Pearson’s correlations were used to determine the relationship and predictiveness between microCT-1µm and microCT-6 µm measures. One-way ANOVA with Tukey’s LSD post-hoc comparisons was used to determine the effects of aging on notochord percentage volume and S.a./V. Statistical analyses were conducted using, Prism 6.0 h (GraphPad Software, La Jolla CA), Excel 2011 (Microsoft, Redmond WA), and Matlab 2015 (Mathworks, Natick MA).

## Electronic supplementary material

 Supplementary Information
