# Supplementary material for: Postnatal Development of the Murine Notochord Remnants Quantified by High-resolution Contrast-enhanced MicroCT
Source: Sci Rep. 2017 Oct 17;7:13361. doi: 10.1038/s41598-017-13446-5 (PMC5645339; doi:10.1038/s41598-017-13446-5)
Supplement: Supplementary file 1 — Supplementary Information [file 41598_2017_13446_MOESM1_ESM.pdf]

Postnatal Development of the Murine Notochord Remnants Quantified by High-resolution Contrast-enhanced MicroCT

Authors: Sameer Bhalla[1], Kevin H. Lin[1], Simon Y. Tang[2,3,4,*]

Affiliations: 1 – Department of Biology
2 – Department of Orthopaedic Surgery
3 – Department of Biomedical Engineering
4 – Department of Materials Science and Mechanical Engineering

Institution: Washington University in St. Louis
660 S. Euclid Ave.
St. Louis, Missouri, 63130, USA

Corresponding Author: Simon Y. Tang, Ph.D.
Assistant Professor
Department of Orthopaedic Surgery
Musculoskeletal Research Center
Washington University, St Louis MO
660 South Euclid Ave, Box 8233
St. Louis, MO 63110
tangs@wudosis.wustl.edu
Phone: (314) 286-2664
Fax: (314) 362-0334

Competing financial interests: The authors declare no competing financial interests.

Supplemental Figures

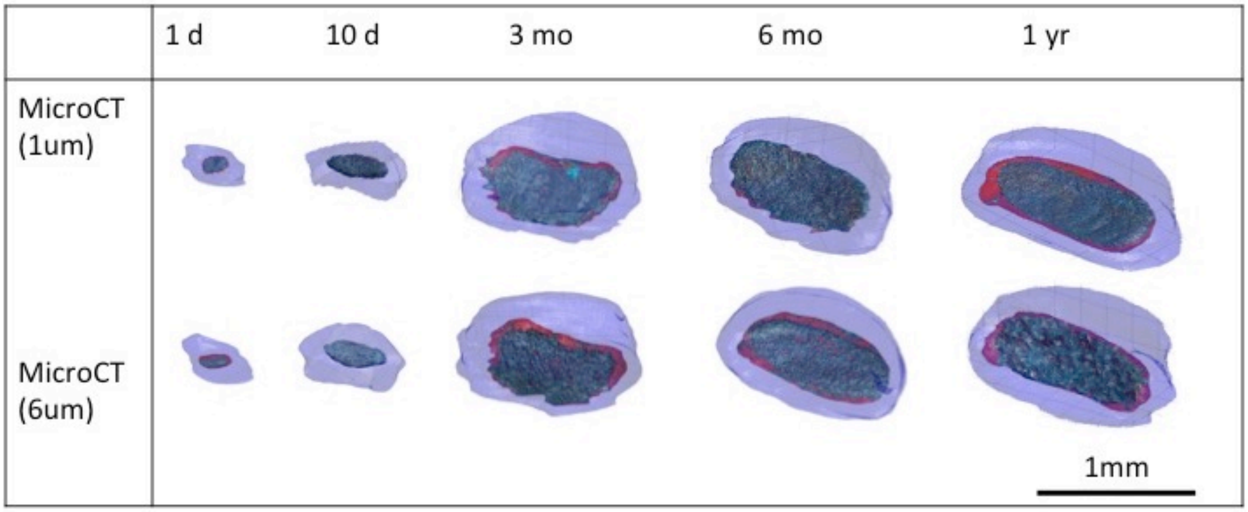

Figure S1: Three-dimensional reconstructions obtained of the intervertebral disc during maturation from both microCT systems depict the structural changes and the growth of each compartment. The annulus fibrosis is depicted as purple, the nucleus pulposus as red, and the notochord tissue as green.
